# Chemical and sensorial investigation of in-mouth sensory properties of grape anthocyanins

**DOI:** 10.1038/s41598-018-35355-x

**Published:** 2018-11-20

**Authors:** M. A. Paissoni, P. Waffo-Teguo, W. Ma, M. Jourdes, L. Rolle, P. -L. Teissedre

**Affiliations:** 1 ISVV, EA 4577 Oenologie, F-33140, Université de Bordeaux, Villenave d’Ornon, France; 2 INRA, ISVV, USC 1366 Oenologie, F-33140, Villenave d’Ornon, France; 3 Dipartimento di Scienze Agrarie, Forestali e Alimentari. Università degli Studi di Torino, Torino, Italy; 4 School of wine, Ningxia University, Yinchuan, Ningxia 750021 P.R. China

## Abstract

Anthocyanins are water-soluble pigments found in the cell vacuoles of fruits and flowers, performing several roles from insects attraction to stress protection. Their antioxidant activity contributes to human health, and consuming plant-derived products provides their higher source in the diet. Although their colour and nutritional features, their contribution to sensory properties of foods has not been widely investigated. In wine, preferences are connected with sensory attributes of colour, aroma, taste, and mouthfeel. In this study, grape anthocyanin extracts (TAE) were fractionated using centrifugal partition chromatography (CPC) and preparative HPLC in three fractions, i.e glucoside (GF), acetylated (AF) and cinnamoylated (CF) anthocyanins. Sensory properties were investigated by chemical analysis, as reactivity towards bovin serum albumin (BSA) and salivary proteins, and in tasting sessions to assess anthocyanins best estimated thresholds (BET) in wine-like solution. Anthocyanins reacted with both BSA and salivary proteins, but to different extents, because higher interaction between salivary proteins and anthocyanins were found. Cinnamoylated anthocyanins are the most reactive to salivary proteins. Tasting sessions suggested an involvement of anthocyanins as in-mouth contributors in wine, since their BETs were 255, 297, 68, and 58 mg/L for TAE, GF, AF, and CF, respectively, and the descriptors reported were astringency and bitterness.

## Introduction

The in-mouth sensory properties of wine are a complex mixture of taste (e.g. bitterness, acidity, sweetness, and saltiness) and mouth-feel sensations, mostly astringency, and flavour. Bitterness and astringency play an important role in the quality of red wine. Bitterness is a taste correlated with the presence of various structured receptors^1^ that are activated by a wide range of molecules, while astringency is a sensation of drying and puckering that is considered to be a mouth tactile response^2^. It is currently accepted that astringent molecules form complexes with salivary proteins due to hydrophobic interactions and hydrogen bonding precipitate the saliva protein, leading to a lack of lubrification in mouth^3,4^. In addition, breakdown of the mouth saliva film is detected by increasing activation of mechanoreceptors, and precipitation of dead cells and other mouth debris increases the feeling of particles in the mouth^5^.

In wine, phenolic compounds are the main class of compounds involved in in-mouth sensory properties, in particular monomeric flavanols and their polymerized forms, usually referred as proanthocyanidins. They are the major compounds influencing wine astringency and bitterness, depending on their concentration, degrees of polymerization and galloylation, B-ring hydroxylation, and their stereochemistry^6–9^. Several methods have been published to quantify tannin astringency based on their ability to react with proteins, such as Serum Bovine Albumin (BSA), gelatine, and salivary proteins^10–12^. While these methods induce the formation of insoluble complex that may precipitate, the interaction between phenolic compounds and protein in soluble complexes has also been reported^13,14^. Thus, astringency is a complex sensation involving several interactive mechanisms that are perceived as intensity and persistence in the mouth. Therefore, overall sub-qualities^15^ can be investigated only by sensorial analysis.

On the other hand, several non-flavanols phenolic compounds have been reported to contribute in in-mouth attributes of wine such as phenolic acids and their derivatives, flavonols, and polymeric pigments formed by the reaction of anthocyanins with flavanols and carboxylic compounds^16,17^. Among them, anthocyanins are a class particularly abundant in grape and wine, since their concentration may reach up to 6 g/Kg^18^ and can be extracted during winemaking. Structurally, anthocyanins are heterosides of an aglycone (anthocyanidin) differentiated among themselves on the number of hydroxylated and methoxylated groups in the anthocyanidin, the nature and the number of bonded sugars in their structure, the aliphatic or aromatic carboxylates bonded to the sugars in the molecule, and the position of this bond. The main anthocyanins present in red winegrapes form *Vitis vinifera* L. are delphinidin, cyanidin, petunidin, peonidin and malvidin, which differ in the B ring substitution, and are present as monoglucoside, acetyl-monoglucoside, caffeoyl-monoglucosides and *p*-coumaroyl-monoglucoside derivatives, where the individual anthocyanidins and esterification can strongly influence their color features, reactivity and stability in wine. Their main role is the contribution to chromatic features of rosé and red wine. They are extracted from grape skins during the first step of the winemaking process and their influence on colour is dependant by the solution pH and by copigmentation. As a fuction of pH, four different forms can be found, e.g. flavylium form (red, pH = 1), quinoidal species (blue, pH = 2–4), and at higher pH as carbinol pseudobase (colourless) and chalcone (yellow). At wine pH (3.0–4.0), these four species coexist, with a prevalence of quinodal species^19^. Copigmentation, a phenomenon in which anthocyanins can form non-covalent linked complexes with other organic compounds, the co-factors, or between anthocyanins themselves (self-association) can stabilize the coloured flavilyum cation. In addition, a change in absorption toward higher wavelenght (bathochromic effect) and higher intensity (hypechromic effect) occurs, and copigmentation is thought to be implicated in up to the 50% of young red wine colour features^20^. On the other hand, once they are extracted, anthocyanins can undergo several reactions with grapes and yeast metabolites to produce new pigments. These reactions produce more complex molecules as long as the wine continues to age, and they are responsible of a minor content of monomeric anthocyanins in aged wines^21–33^ and Table S1 provides an overview of grape and wine contents of pigmented materials. This process is considered to be responsible for the changing sensory properties of wine during ageing, such as the shift of colour from bluish-red to orange and the increasing smoothness of astringency for the complexation of monomeric and polymeric flavanols.

Although the role of anthocyanins in wine colour has been widely investigated, their contribution to in-mouth sensory properties is still controversial. Several studies have attempted to explain their involvement in taste and mouthfeel properties, but without any clear consensus. Anthocyanins are reported to have a “mild taste”^34,35^, and increasing astringency, in particular sub-qualities as “fine grain”^13,36,37^. Later, Gonzalo-Diago *et al*.^38^ found the acetylated and coumaroylated anthocyanins contributed to both astringency and bitterness. The chemical determination of astringency as interaction with salivary protein was achieved with glucoside anthocyanins. Notably, malvidin-3-*O*-glucoside was found to form soluble complexes with salivary proteins^13^ and to activate TAS2R7 bitterness receptor^39^. Anyway, Vidal *et al*.^40^ found no differences either in model wine added with glucosides or coumaroylated anthocyanins or in slightly unbuffered ethanolic solution (5%), thereby confirming the in-mouth sensation reported previously as impurities in the isolated fractions.

To date, obtaining pure anthocyanin samples in sufficient quantity has been a problem in characterizing their sensory properties. Centrifugal partition chromatography (CPC) is a liquid-liquid separation technique that allows different solvents to be used as stationary and mobile phase as long they are immiscible, and which can be adapted for injecting several grams of raw extract. Liquid-liquid separation of anthocyanins has been successfully achieved by multi layers and high speed countercurrent chromatography (MLCCC and HSCCC), and centrifugal partition chromatography (CPC) of different capacity (up to 5L) from fruit extracts and in particular from grape skins, marcs, and wines^41–45^.

The aim of this study was to isolate anthocyanins classes present in wine grapes and to evaluate the sensoactive features by chemical and sensorial analysis. To obtain purified glucoside, acetylated and cinnamoylated (as mix of caffeoylated and coumaroylated derivatives), *Vitis vinifera* L. c.v. Nebbiolo and Barbera were extracted from skin and fractionated using CPC and preparative HPLC techniques. These two varieties were chosen because they have different anthocyanin profiles, which we expected to provide a different degrees of fractionation. The reactivity of the extract and fractions toward proteins as a marker of astringency was tested by adapting BSA and salivary protein precipitation methods. Sensory analysis was performed in addition to chemical investigation, and a in-mouth detection threshold was estimated for total anthocyanins extract, and for glucosides, acetylated, and cinnamoylated anthocyanin classes.

## Results

### Anthocyanins extraction and purification

**Figure 1:**
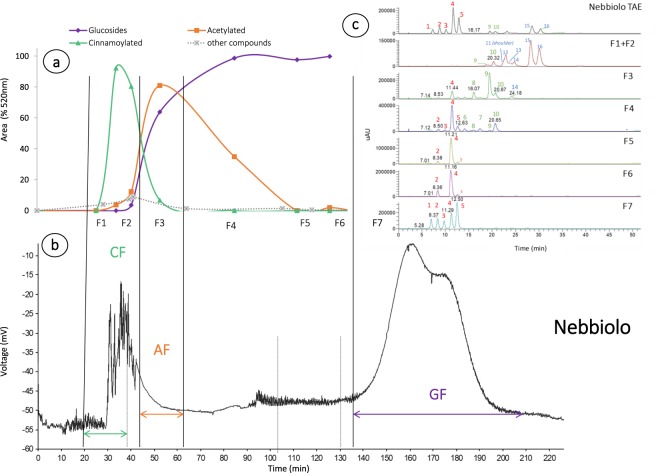
Nebbiolo CPC separation results. (**a**) 1L-CPC concentration of glucoside, acetylated and p-coumaroylated anthocyanins and other compounds as percentage at 520 nm. (**b**) 200ml-CPC chromatogram at 280 nm and corresponded collected fraction CF = cinnamoylated fraction, AF = acetylated fraction, and GF = glucoside fraction. (**c**) HPLC-UV chromatograms of Nebbiolo total anthocyanins extract (TAE) and CPC fractions (λ = 520 nm). Peak numbers are reported in Table 1.

**Figure 2:**
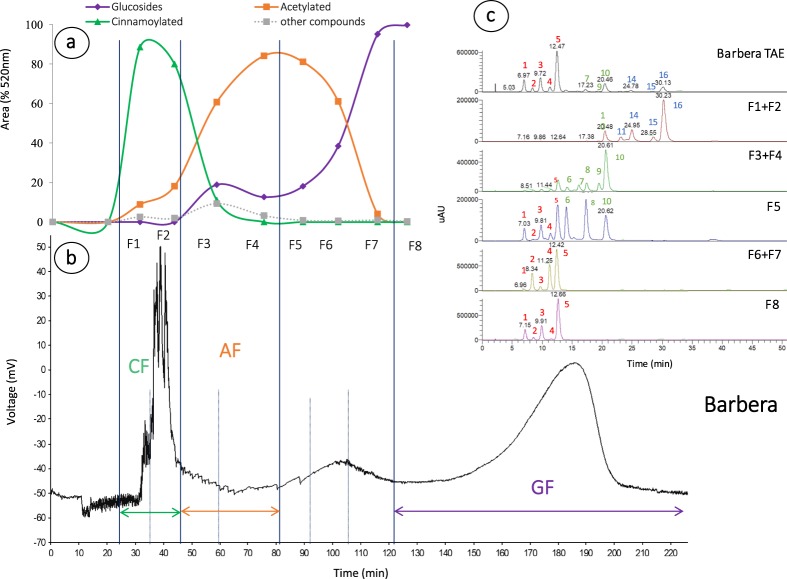
Barbera CPC separation results. (**a**) 1L-CPC concentration of glucoside, acetylated and *p-*coumaroylated anthocyanins and other compounds as percentage at 520 nm. (**b**) 200 ml-CPC chromatogram at 280 nm corresponded collected fraction CF = cinnamoylated fraction, AF = acetylated fraction, and GF = glucoside fraction. (**c**) HPLC-UV chromatograms of Barbera total anthocyanins extract (TAE) and CPC fractions (λ = 520 nm). Peak numbers are reported in Table 1.

Extraction of grape skin anthocyanins produced compounds of 7.2% and 5.8% for Barbera and Nebbiolo, respectively, on the total skin powder weight (w/w). As expected by the total anthocyanins concentration of the variety, Barbera produced higher quantity than Nebbiolo^18,46^. The latter, has a particular anthocyanin profile because it is a disubstituted prevalent variety, so peonidin and cyanidin derivatives are particularly abundant accounting for the 51.67% of the total anthocyanins. Barbera is, as usual in *Vitis vinifera* L, a malvidin-prevalent variety, so trisubstituted anthocyanins accounted for the 90% of all anthocyanins (chromatographic profiles are reported in Figs 1c and 2c, peak numbers and corresponding molecules are reported in Table 1). Regarding esterification, 79.7% and 72% were glucoside, whereas 8% and 14.3% were acetylated and there were 12.3% and 13.7% *p*-coumaroylated and caffeoylated derivatives for Nebbiolo and Barbera, respectively. Purity of total anthocyanins extracts (TAEs) was calculated from the peak visible at 520 nm and 280 nm chromatograms and is reported as percentage. It was higher than 95% for both Nebbiolo and Barbera. Fast 4-hour extraction partially avoided the extraction of other phenolic compounds which may interfere with in-mouth chemical and sensorial analysis, especially oligomeric and polymeric flavanols. Regarding monomeric flavanols, neither catechin nor epicatechin were detected in TAEs. The main impurities in the extract were flavonol that were detected at 365 nm.

**Table 1:** Composition of Barbera total anthocyanins extract and derived glucoside, acetylated and cinnamoylated fractions expressed as percentage on the HPLC chromatogram at 520 nm. Peak numbers correspond to chromatogram in Figs 1c, 2c and 3a–d.

Peak	Compounds	Composition			
		Total Anthocyanins (%)	Glucoside Fractions (%)	Acetylated Fractions (%)	Cinnamoylated Fractions (%)
1	Delphinidin-3-*O*-glucoside	10.7	12.96		
2	Cyanidin-3-*O*-glucoside	3.19	13.58		
3	Petunidin-3-*O*-glucoside	12.73	10.82		
4	Peonidin-3-*O*-glucoside	4.16	22.04		
5	Malvidin-3-*O*-glucoside	43.24	40.6		
6	Delphinidin-3-*O*-acetylglucoside	1.25		2.34	
7	Cyanidin-3-*O*-acetylglucoside	0.45		3.73	
8	Petunidin-3-*O*-acetylglucoside	2.4		7.7	
9	Peonidin-3-*O*-acetylglucoside	0.45		17.57	
10	Malvidin-3-*O*-acetylglucoside	10.38		66.55	
11	Delphinidin-3-*O*-coumaroylglucoside	0.33			10.56
12	Malvidin-3-*O*-caffeoylglucoside	0.56			4.12
13	Cyanidin-3-*O*-coumaroylglucoside	nd			2.13
14	Petunidin-3-*O*-coumaroylglucoside	1.79			10.86
15	Peonidin-3-*O*-coumaroylglucoside	0.65			24.77
16	Malvidin-3-*O*-coumaroylglucoside	7.77			47.56

A first attempt at separation was carried out using a 200 ml CPC according to an already published method^41^. Since the separation was satisfactory, the system was then applied to a larger apparatus (1L). Normal-phase CPC was conducted, so the stationary phase was constituted by the aqueous and the mobile phase corresponding to the organic solvents of low (B1) and high polarity (B2) in gradient. By doing so, the less polar cinnamoylated (*p*-coumaroylated and caffeoylated) anthocyanins eluted first during the isocratic phase of solvent B1, followed by acetylated, eluted with the gradient up to 50% of solvent B2, and then glucosides during the gradient to 100% B2. Figures 1b and 2b shows the chromatogram of 200 ml CPC for Nebbiolo and Barbera, respectively. The 1L-CPC chromatogram is equivalent, although it finishes at 140 minutes since it was stopped after acetylated separation, and glucosides were collected with extrusion of the stationary phase. The fractions collected in CPC 1L are shown in Figs 1a and 2a, reported as percentage of cinnamoylated (caffeoyl and *p*-coumaroyl derivatives), acetylated, and glucosilated forms found in each fraction by 1L CPC separation, for Nebbiolo and Barbera, respectively. Regarding glucoside anthocyanins, separation was in accordance with previous reports since they eluted according to the hydroxylation/methoxylation substitution: thus, cyanidin and peonidin, which are disubstituted anthocyanins, are eluting first than delphinidin, petunidin, and malvidin, which are trisubstituted^41,43^. This elution, did not provide fractions that differed between varieties. Only peonidin-3-*O*-glucoside in Nebbiolo could be extracted as almost pure compound (F5-F6, Fig. 1c). CPC allowed for good separation depending on the esterification of the glucoside moiety, although it was not able to completely avoid the presence of other derivatives. Notably, the most abundant anthocyanin, i.e. malvidin, is present in acetylated fraction as *p*-coumaroylated form, and in acetylated as its glucoside form. The great advantage of this technique is the quantity obtained and in particular the possibility to collect sufficient amount of acetylated and cinnamoylated derivatives by extruding the most abundant glucosides. For Nebbiolo (Fig. 1c), *p*-coumaroylated and caffeoylated anthocyanins eluted in the first two fractions, and acetylated were eluted in fraction 3, 4 and 5. Although glucosides were abundant from F4, particularly in Nebbiolo, given the abundance of di substituted glucoside which eluted first. Finally, F1 and F2 were collected as a cinnamoylated fraction (CF), F3 as an acetylated fraction (AF) and F8 as a glucosides fraction (GF) which corresponded to the 12.98%, 3.86%, and 83% of the total amount injected. Separation was similar for Barbera (Fig. 2c) where F1 and F2 were collected as CF, F3 and F4 as AF, and F8 as GF, but higher proportion of esterified anthocyanins was found accounting 31.82%, 10.84% and 57.33% for CF, AF and GF, respectively.

Since there were no interesting differences between the two varieties, the fractions collected from them were mixed together for chemical and sensory analysis, producing a final amount of 820.8 mg of CF, 303.3 mg of AF and 3016 mg of GF.

**Figure 3:**
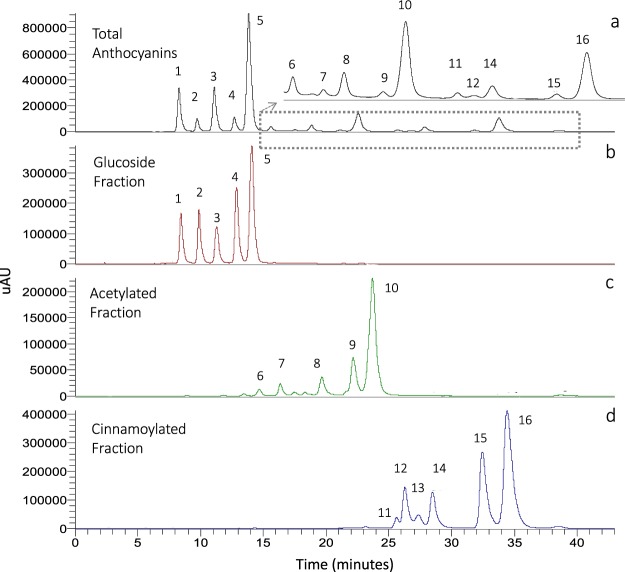
Chromatogram of HPLC-DAD analysis at 520 nm of anthocyanins used for chemical and sensorial analysis. (**a**) Total anthocyanins extract of Barbera, (**b**) glucoside fraction, (**c**) acetylated, and d) cinnamoylated fractions of anthocyanins. Corresponding molecule identifications are reported in Table 1.

TAE impurities (i.e. other phenolic compounds detected at 280 nm, mainly flavonols) were eluting in the beginning of the separation, in particularly in CF and AF fractions, with 60.86% and 66.7% of anthocyanins detected respectively, whereas high purity was achieved for GF directly from CPC extrusions (98.55%). Therefore, a preparative HPLC separation was performed to remove the impurities and to isolated anthocyanins not belonging to the same derivatives class for F4 and F5 for Nebbiolo and F5 for Barbera, in order to recover acetylated anthocyanins. Both F1 and F2 (CF) were purified to extrude impurities. The final purity achieved was 91% for CF and 85% for AF. Purification of acetylated fractions was very difficult because of the presence of peaks at 280 nm co-eluting with anthocyanins, and above all to a loss of acetic acid moiety during fraction evaporation which gave the respective simple glucoside anthocyanins. Therefore, AF purification was conducted several times, which leaded to a great loss of compounds. The level of 85% purity of acetylated fraction was reached, and where a 5% of impurities corresponded to their relative glucosides. Final fractions obtained are shown in Fig. 3b–d.

### Chemical evaluation of astringency

While there are numerous methods to quantifiy astringency in wine and tannin extracts, there are fewer for anthocyanins. When the saliva test was coupled with MALDI-TOF to detect anthocyanin glucosides interaction, the proline protein (PRPs) and histatin chromatographic profiles were different with or without anthocyanins^13^. In particular, the decrease of PRPs fraction of saliva in the presence of anthocyanins leads to supposition of the formation of precipitable complexes. The strength of the affinity between malvidin-3-*O*-glucoside and PRPs, evaluated as dissociation constant (*K*_D_), was assessed by STD-NMR spectroscopy with success^13^. Also, binding between anthocyanins and human serum albumin (HSA) has been reported, and *K*_D_ at different pH was determined^47^. Therefore, saliva and bovine serum albumin (BSA) were assessed on total anthocyanins and fractions by common methods used on other classes of phenolic compounds.

To conduct the experiment of chemical and sensory analysis, CPC fractions combined by Nebbiolo and Barbera were taken, and Barbera TAE alone was used as total anthocyanins extract, since its anthocyanins profile is similar to that of most *Vitis vinifera* cultivars and therefore can be more representative of wine anthocyanins profile.

**Figure 4:**
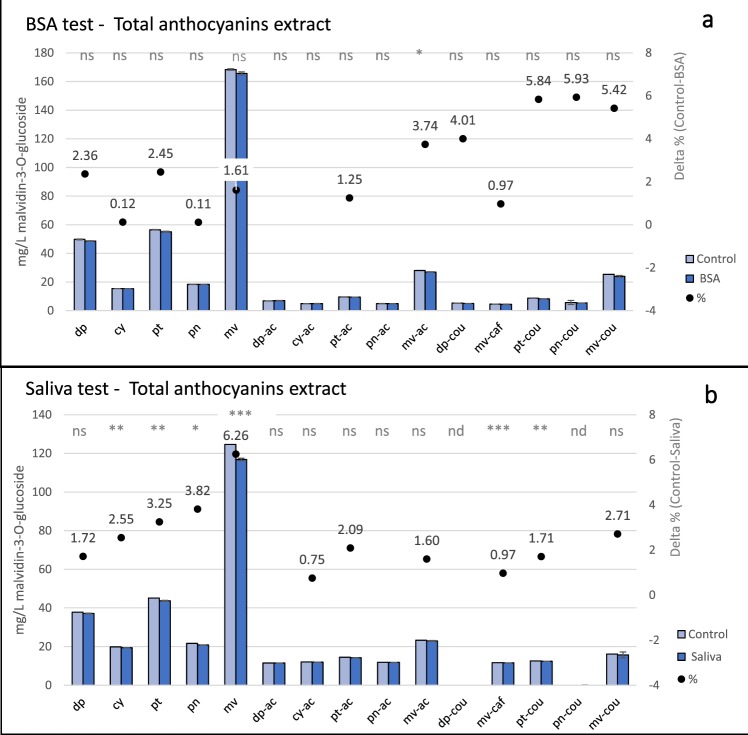
BSA (**a**) and Saliva (**b**) tests results on total anthocyanins extracts. All data are expressed as average value ± standard deviation (*n* = 3). Sign: *, **, ***, and ns indicate significance at *p* < 0.05, 0.01, 0.001, and not significant, respectively, for difference between each identified compound for control and treated samples. Delta between treated (BSA and Saliva) and control (•) is reported as percentage for each compound, not reported delta are ≤ 0. Legend: dp = delphinidin-3-*O*-glucoside, cy = cyanidin-3-*O*-glucoside, pt = petunidin-3-*O*-glucoside, pn = peonidin-3-*O*-glucoside, mv = malvidin-3-*O*-glucoside, dp-ac = delphinidin-3-*O*-acetylglucoside, cy-ac = cyanidin-3-*O*-acetylglucoside, pt-ac = petunidin-3-*O*-acetylglucoside, pn-ac = peonidin-3-*O*-acetylglucoside, mv-ac = malvidin-3-*O*-acetylglucoside, dp-cou = delphinidin-3-*O*-coumaroylglucoside, mv-caf = malvidin-3-*O*-caffeoylglucoside, pt-cou = petunidin-3-*O*-coumaroylglucoside, pn-cou = peonidin-3-*O*-coumaroylglucoside, mv = malvidin-3-*O*-coumaroylglucoside.

BSA and Saliva test were first applied to Barbera TAE. BSA showed a significant difference only for malvidin-3-*O*-acetylglucoside (Fig. 4a, −3.74%, *p* < 0.05). Saliva test detected a significant difference between the saliva and control samples especially for glucosides (Fig. 4b, cyanidin and petunidin −2.55% and −3.25%, respectively *p* < 0.01; peonidin −3.82% *p* < 0.05; malvidin −6.26%, *p* < 0.001) and cinnamoylated anthocyanins (for *p*-coumaroylated petudin −1.71%, *p* < 0.01; for caffeoylated malvidin −0.97%, *p* < 0.001). Higher reactivity towards saliva than BSA was also found as sums of anthocyanins, since only saliva treated samples had lower values than control (−3.52%, *p* < 0.01). The first impression is that since glucosides are the most abundant class in the extract, that they may mask the individual behaviour of the derivatives. The difference between BSA- and saliva-treated samples and their respectively untreated controls (delta) was correlated with initial concentration of individual anthocyanins (n = 12, *R*^2^ Spearman = 0.75, *p* < 0.01 and *R*^2^ Spearman = 0.92, *p* < 0.001 for BSA and saliva, respectively).

**Figure 5:**
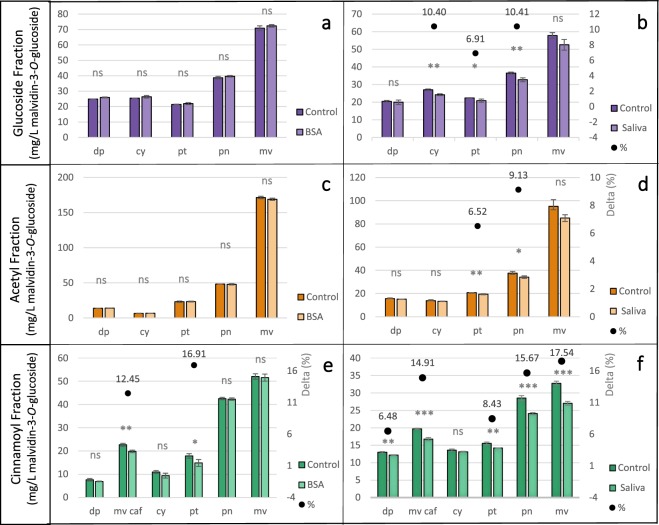
BSA and Saliva tests results on glucoside fraction (**a**,**b**, respectively), acetylated (**c**s**d**, respectively), and cinnamoylated (e and f, respectively) fractions. All data are expressed as average value ± standard deviation (*n* = 3). Sign: *, **, ***, and ns indicate significance at *p* < 0.05, 0.01, 0.001, and not significant, respectively, for difference between each identified compound for control and treated (BSA and Saliva) samples. Delta between treated (BSA and Saliva) and control (•) is reported as percentage for each compound, deltas is for significantly different compounds reported only. Legend: for (**a**,**b**), dp = delphinidin-3-*O*-glucoside, cy = cyanidin-3-*O*-glucoside, pt = petunidin-3-*O*-glucoside, pn = peonidin-3-*O*-glucoside, mv = malvidin-3-*O*-glucoside; for c and d, dp = delphinidin-3-*O*-acetylglucoside, cy = cyanidin-3-*O*-acetylglucoside, pt = petunidin-3-*O*-acetylglucoside, pn = peonidin-3-*O*-acetylglucoside, mv = malvidin-3-*O*-acetylglucoside; and for e and f, dp = delphinidin-3-*O*-coumaroylglucoside, mv-caf = malvidin-3-*O*-caffeoylglucoside cy = cyanidin-3-*O*-coumaroylglucoside, pt = petunidin-3-*O*-coumaroylglucoside, pn = peonidin-3-*O*-coumaroylglucoside, mv = malvidin-3-*O*-coumaroylglucoside.

Therefore, to avoid any concentration effect, analysis was then carried out on CPC fractions (Fig. 5). Altough a coloured precipitation occurred with BSA, no significant differences were found in total GF and AF, whereas a significant decrease in total CF was detected (−5.75%, *p* < 0.05), i.e caffeoylated malvidin and *p*-coumaroylated petunidin decreased by 12.45% (*p* < 0.01) and 16.91% (*p* < 0.05), respectively (Fig. 5e). Greater differences were detected with the saliva test: the concentration of anthocyanins is reduced in all samples, with −8.53% and −9.48% (*p* < 0.05) for GF and AF, respectively, and −12.82% (*p* < 0.001) for CF. Figure 5b shows that cyanidin and peonidin were decreased of −10.4% and −10.41% respectively (*p* < 0.01), and petunidin of −6.91% (*p* < 0.05) in GF. Malvidin, the most abundant glucoside, was reduced of 9.17% (*p* = 0.054). Among the acetylated form (Fig. 5d), petunidin and peonidin were decreased by −6.52% (*p* < 0.01) and −9.13% (*p* < 0.05) and malvidin by 10.59% (*p* = 0.053). Highly significant differences were found for CF for all compounds except cyanidin (Fig. 5f): *p*-coumaroylated delphinidin and petunidin were decreased by −6.48% and 8.43% with respect to the control (p < 0.01), whereas *p*-coumaroylated peonidin and malvidin were decreased by 15.67% and 17.54% (p < 0.001). In addition, saliva-treated caffeoylated malvidin decreased by −14.91% (p < 0.001) with respect to the control.

Saliva more reliably reproduces the in-mouth anthocyanin behaviour than BSA, since it contains the proline-rich proteins (PRPs) responsible for complexes precipitation, whereas BSA is a common protein substitutive that may not react to form precipitable complexes with anthocyanins. In fact, even if the coefficient of variation between replicates (c.v. < 5%) was achieved with an adapted BSA test, a large standard deviation was found among BSA-treated samples, thus confirming the hypothesis that affinity between the protein and anthocyanins is poor. It was previously reported that small phenolic compounds do not form insoluble complexes with protein^13,14^. In our study, a red precipitate was found in BSA-added samples, but in most of the cases there was not significant difference. Moreover, an interaction can occur between proteins and anthocyanins and lead to soluble compounds in wine-like solution, but it was not detectable with the method used. Therefore, a qualitative but not quantitative estimation of anthocyanins-protein interaction is possible. Regarding saliva-anthocyanins interaction, Ferrer-Gallego *et al*.^13^ reported the formation of soluble complexes between malvidin-3-*O*-glucoside and a peptide sequence of histatin and proline-rich proteins. The latter, which can be considered high molecular weight proteins among salivary protein fractions^13^, are thought to form insoluble complexes and therefore to precipitate with several polyphenols^3^. In our study, the CF fraction was the most reactive, perhaps due to the known reactivity of coumaric and caffeic acids with salivary protein^48^ and their involvement in wine astringency^16^.

The correlation between anthocyanidin substitutes (i.e delphinidin, cyanidin, petunidin, peonidin, and malvidin) and their delta (difference between control and treated, as marker of the magnitude of the interaction leading to a precipitation) was not significative, except for cyanidin (*R*^2^ Pearson = 0.99, *p* < 0.01). Regarding flavanols (i.e. catechin and gallocatechin), the substitution of B ring strongly influences astringency, in particular the presence of two or three hydroxyl groups, since di-hydroxylated compounds lead to negative sensory attributes of astringency, such as “dry”, “rough”, and “unripe”, whereas tri-hydroxylated compounds are correlated with the positive attributes “velvety”, “smoothness”, and “viscosity” that arise from different interaction among the molecules and protein^49^. Further studies, should be conducted on individual anthocyanins since the concentration effect can mask the difference in the reactivity of individual anthocyanins to protein depending on the B ring substitution, owing to the presence of the methyl group in peonidin, petunidin, and malvidin. On the other hand, when the CPC fractions were treated with salivary protein, the glucoside esterification was highly correlated with the precipitate concentration (*R*^2^ Pearson = 0.939, *p* < 0.05, *R*^2^ Pearson = 0.999, *p* < 0.001, and *R*^2^ Pearson = 0.996, *p* < 0.001, for glucoside, acetylated, and cinnamoylated, respectively). Altogether, our results show that reactivity of anthocyanins is mainly dependent on glucoside acylation, with *p*-coumaroyl and caffeoyl moieties increase the interaction between anthocyanins and salivary proteins.

### Sensory analysis of extract and fractions

As described above, in-mouth properties involve various parameters of which astringency is only one. In addition, bitterness can be tested by using receptors, as done successfully for malvidin-3-*O*-glucoside^39^. However a limitation of this technique is that only 1% ethanol content can be used owing to its cellular toxicity, so true wine condition are not completely reproduced. To understand whether anthocyanins can be detected in wine, we performed a sensory detection thresholds test for the Barbera total anthocyanins extract (TAE) and the fractions cinnamoylated (CF), acetylated (AF), and glucoside (GF). A wine scale range and wine model solution were chosen to estimate the detection threshold. The best estimate threshold method (BET) was used since it is very difficult to obtain a sigmoid curve in taste threshold. Several factors should be taken into account, especially variability in tasters^50^. The quality and quantity of the multiple cellular mechanisms associated with bitter taste varies considerably from one person to another. Moreover, receptors saturation during tasting with several presentations can occur.

Detection threshold test results are shown in Table 2, with the concentrations used. Sensory results were in agreement with chemical data, since CF, which was the most reactive towards protein, was also the fraction with the lowest perception threshold (BET = 58 mg/L), followed by AF (BET = 68 mg/L). Moreover, the higher perception threshold of anthocyanins glucosides alone (BET = 297 mg/L) than TAE (BET = 255 mg/L) suggested that the presence of a percentage of acetylated and cinnamoylated anthocyanins on the total extract had a higher impact on sensory properties, thereby lowering the BET of total anthocyanins extract. Judges were asked to express one (or more) in-mouth descriptor(s) that helped them to discriminate the samples, and Table 2 shows descriptors only for those judges who correctly discriminate the sample over the BET. The common descriptors were astringency and bitterness for all the anthocyanins tasted, and saltiness was reported for glucoside and total extract. Saltiness was described as a tingling sensation on the tongue. Taster were not trained for astringency sub-qualities for the detection threshold test, so the “saltiness” descriptor could be misunderstood as a mouthfeel sensation such as irritation or the particulate in mouth of astringency, as proposed by Gawel *et al*.^15^. This is in accordance with previous studies which reported a “mild taste” of anthocyanins in solution and in particular an increase in astringency (particularly “fine grain” attribute) descriptor^36,37^. Previous research also estabilished a relationship between the presence of anthocyanins (especially acetylated and *p*-coumaroylated) and the perceived bitterness of wine^38^. Under our conditions, all the solutions tasted were described as bitter tasting. It seems that anthocyanins are involved in in-mouth sensory properties at wine concentration and that their influence depends on the esterification of glucosides.

**Table 2:** ^*^BET (Best estimated threshold) of total anthocyanins extract of Barbera and of CPC Fractions, with tasted concentrations and correspective correct answers. ^†^Descriptors are reported only for correct answers for concentrations over the BET.

Group	BET^*^ (mg/L)	Log_10_ BET	Log_10_ St. Dev.	Concentration (mg/L)	Descriptors^†^ (n)
Total Anthocyanins (mg/L)	255	2.41	0.75	2000	Astringency (8); Bitterness (8); Saltiness (5)
				1000	Astringency (7); Bitterness (6); Saltiness (3)
				500	Astringency (6); Bitterness (5); Saltiness (2)
				250	
				125	
				62.5	
Glucosides (mg/L)	297	2.47	0.50	1000	Astringency (10); Bitterness (5); Saltiness (4)
				500	Astringency (6); Bitterness (8); Saltiness (4)
				250	
				125	
				62.5	
				31.125	
Acetylated (mg/L)	68	1.81	0.34	100	Bitterness (7); Astringency (4)
				50	Bitterness (9); Astringency (9)
				25	
				12.5	
				6.25	
				3.125	
Cinnamoylated (mg/L)	58	1.76	0.42	100	Bitterness (4); Astringency (3)
				50	Bitterness (4); Astringency (4)
				25	
				12.5	
				6.25	
				3.125	

## Discussion

Anthocyanins are well-known for their contribution to wine colour and chromatic features, and several vineyard and winemaking strategies are exploited to ensure the maximum anthocyanins accumulation and extraction for improve wine’s visual quality. On the other hand, understanding of their influence on in-mouth sensory properties is only partial, in particular regarding astringency, mouthfeel attributes, and bitter taste. Wine in-mouth sensation variability is recognized to be strongly connected to flavanols concentration and characteristics, however several molecules can contribute to in-mouth sensations and implicate wine in-mouth complexity. Full understanding of these different factors can help in the definition of winemaking strategy. Therefore, in this study, grape anthocyanins were extracted from skins and fractionated in classes depending on their substitution, i.e. glucoside, acetylated and cinnamoylated, by a combination of liquid–liquid chromatography (CPC) and preparative-HPLC. Yield and purity were of sufficient quality and quantity to investigate their sensory properties, in particular regarding glucoside anthocyanins whereas acetylated and cinnamoylated anthocyanins contained some impurities. These compounds, that were mainly detected at 365 nm, were considered belong to flavonols classes, which are involved in in-mouth sensory properties and therefore may have influenced sensory analysis results. Taste detection thresholds of these compounds as previously reported^35^ and trace level detected lead to exclude this hypothesis. Both chemical and sensory analyses were performed. Additionally, this is the first time that acetylated anthocyanins have been tasted, and the interaction of acetylated and cinnamoylated anthocyanins with protein assessed. Anthocyanins reacted with both BSA and salivary protein, but to different extents, as the saliva test gave higher response between anthocyanins and salivary protein. Importantly, the saliva test revealed a significant reduction of anthocyanins, both in the total extract and when fractionated in glucoside, acetylated, and cinnamoylated. The latter in particular is the most reactive to salivary protein. These results are confirmed by sensorial analysis carried out by detection threshold test. Best estimated threshold (BET) of anthocyanins were in wine range scale, and acetylated and cinnamoylated thresholds were below the glucoside threshold. This was confirmed by the lower BET of total extract compared to the glucoside fraction alone. Therefore, anthocyanins can be detected as contributors to in-mouth properties, and the degree of their involvement is related to their acylation. Indoubitably, anthocyanins concentration in wine must be considered: BETs concentration as hereby reported are founded in young and anthocyanins-rich wines, therefore the presence of other well-known eliciting compounds, such as monomeric and polymeric flavanols, is still to assume as the major contribution to wine astringency and bitterness. Interaction between anthocyanins and other phenolic class compounds are reported in studying wine colour, such as copigmentation. These non-covalent reactions may influence affinity of both cofactor and anthocyanins for salivary protein, as recently reported by Soares *et al*.^51^ for malvidin-3-*O*-glucoside and epicatechin. Therefore, in addition to the individual compound concentration, the interaction with other sensoactive compounds is also relevant from several points of view, including the direct interaction with salivary proteins or the bitter receptors, the interaction between the compounds themselves, and the competition to elicite the sensation. Further sensory analysis should be carried out with a panel trained in mouthfeel descriptors to investigate the in-mouth descriptors of anthocyanins in more complex solutions. In particular, evaluation of pH and ethanol content, and the interaction with other taste compounds will be useful in order to achieve a deeper understanding of the wine in-mouth complexity.

## Methods

### General Information

#### Chemicals

Distilled water was obtained from an ELGA system, and Milli-Q (Millipore) water was prepared using a Sarterius-arium 611 system. All solvents were HPLC grade, in detail: methanol, acetonitrile, and ethyl acetate were 99.9% and 1-buthanol was 99.8%. Formic acid and trifluoroacetic acid were ≥95% and 99%, respectively. They were purchased from Prolabo-VWR (Fontenays/Bois, France).

#### Ethical Permission

The ethical committee of Laboratory Research Unit USC 1366 Board, Institut des Sciences de la Vigne et du Vin of University of Bordeaux (ISVV) approved the study for saliva collection of volunteers. All participants signed an informed consent form with type of research, voluntary participation and saliva collection protocol by spitting.For sensory analysis, participants were volunteers and signed an informed consent form with type of research, voluntary participation and agreement to taste of extracts produced as in protocol described in section “Total anthocyanins extracts and samples purification”.

### Apparatus and Analytical Methods

#### Centrifugal Partition Chromatography (CPC)

The 200 mL CPC was an FCPC 200 provided by Kromaton Technologies (Saintes-Gemmes-sur-loire, France), consisting of a rotor (20 circular partitions disks, total volume capacity of 204 ml; 1320 partitions cells). High-pressure gradient pump (Gilson 321-H1) and high-pressure injection valve (21 mL loop, Rheodyne) were used for the gradient. The rotor ran at 1000 rpm, at 3 mL/min flow rate. Chromatogram was checked by a Kromaton UV-Vis detector at 280 nm. Fraction were collected every 3 minutes for each tube by a Gilson 204 fraction collector and analysed in analytical HPLC-DAD system. The system allowed the injection of 100 mg for each run in 10 mL of lower phase. Retention of stationary phase was calculated as 74.4%. The 1L centrifugal partition chromatography (CPC) apparatus was an FCPC 1000 provided by Kromaton Technologies (Saintes-Gemmes-sur-Loire, France). It consisted of a rotor (45 circular partition disks; total column capacity of 940 mL; 1440 partition cells), a binary high-pressure gradient pump (Gilson 321-H1), a high-pressure injection valve (50 mL sample loop, Rheodyne) and a Kromaton UV–vis detector. Fractions were collected manually checking the UV-Vis signal at 280 nm and 520 nm. Anthocyanins extract (maximum 2.5 g) were dissolved in lower phase (40 mL) and filtered prior injection (0.45 um). CPC method was the compatible with the system described above, the rotor was running at 1000 rpm, and flow rate was 15 mL/min. Retention of stationary phase was calculated as 76.1%.

#### Preparative High-Performance Liquid Chromatography (PREP-HPLC)

PREP- HPLC was performed on a Varian LC machine consisting of a Prostar 210 two-way binary high-pressure gradient pump, a 2 mL loop and a Prostar 325 UV/Vis detector, recording at 520 and 280 nm. The column use was a Nucleosil C18 (21 × 250 mm, 5 µm) and the mobile phase consisted of acidified acetonitrile (Eluent B) and acidified water (Eluent A), both containing 0.1% TFA. The flow rate was 10 mL/min and the gradient was from 15% to 45% of B in 35 minutes, followed by 7 minutes of 100% B and reconditioning at 15% B for 7 minutes. For each injection, 40 mg of fraction compounds were dissolved in 250 µL 50:50 (v/v) methanol/water acidified with 0.1% TFA and manually injected into the system.

#### Analytical High-Performance Liquid Chromatography-Diode Array Detection (HPLC-DAD)

Anthocyanins extracts and fractions analysis were performed on a Thermo-Finnigan Accela HPLC system consisting of an autosampler (Accela autosampler), pump (Accela 600 Pump), and diode array detector (Accela PDA Detector) coupled to a Finnigan Xcalibur data system. Separation was performed on a reversed phase Agilent Nucleosil C18 (250 mm × 4 mm, 5 μm) column. Gradient consisting of water/formic acid (95:5, v/v) (solvent A) and acetonitrile/formic acid (95:5, v/v) (solvent B) was applied at a flow rate of 1 ml/min. Method was slightly modified from Chira (2009) as follow: 10–23% B linear from 0–16 minutes, 23–28% B in 19 minutes, 28–100% B in 6 minutes, 100% isocratic B for 5 minutes, 100% B gradient to initial condition for 6 minutes and re-equilibration of the column for 3 min under the initial gradient conditions. Purity was checked as 520/280 nm detectable peaks. Peaks were previously identified with MS injection^52^ and quantification was done on malvidin-3-*O*-glucoside (Sigma–Aldrich, Saint Quentin Fallavier, France) calibration curve.

#### Total anthocyanins extracts and samples purification

50 kg of Nebbiolo and Barbera grapes were harvested in Alba (Piedmont, Italy) at full ripeness, cutted in small cluster (5-6 berries each), collected in small boxes of 600 g each and stored at −20 °C. For skins processing, one small box at time was taken and skins were removed with a laboratory spatula by frozen berries and washed with water to remove potentially pulp residues. Skins were then freeze-dried for two days and grounded to powder in a ball grinder. Skins powders were stored at −20 °C, until extracted. For Nebbiolo, a total of 5161 g of berries were peeled, giving 573 g of fresh grape skins and final lyophilized skins weight was 197.5 g. For Barbera, 5174 g of berries were peeled, giving 536 g of fresh skins and final lyophilized skins weight was 210.1 g. Extraction was performed on 100 g of skin powder in 1L acidified methanol as solvent (0.1% TFA) for two hours two times under stirring. The recovered solvent was filtered to avoid particulate, evaporated and freeze dried. The anthocyanins extract was cleaned from acids and sugars through solid phase extraction (SPE) using Amberlite XAD 16 resin (Sigma–Aldrich, Saint Quentin Fallavier, France). A large-scale column was filled with 1 Kg of resin and samples were washed with acidified water (0.1% TFA) until the eluate was clear (around 2 bed volumes). Anthocyanins were then recovered with acidified methanol (0.1% TFA), evaporated and freeze-dried. The resulting powder was used to CPC fractionation and it is the so-called total anthocyanins extract (TAE) and was stored at −20 °C until needed. Purity and composition of Nebbiolo and Barbera TAEs were checked with HPLC-DAD system, slightly modified from Chira^52^.

Two different CPC equipments were used, 200 mL CPC was used to carried out method improvement and a 1L CPC to obtain the powder designated to sensorial and chemical analysis (details of CPC apparatus are described in “Apparatus and analytical method” section). The CPC system were adapted from Renault *et al*.^41^: apparatus was working in ascending mode where lower phase, as stationary, was composed by Ethyl Acetate:Butanol:Water 5:5:90 (v/v/v), whereas a gradient of two mobile phase was applied using two solvent B system Ethyl Acetate:Butanol:Water 770:150:80 (v/v/v) as initial mobile phase (B1) and Ethyl Acetate:Butanol:Water 400:460:140 (v/v/v) as final mobile phase (B2). The gradient was: 30 minutes 100% of B1, from 100% B1 to 50%B1/50% B2 in 90 minutes, 30 minutes 50%B1/50%B2, to 100% B2 in 60 minutes, and 100%B2 for 90 minutes. Regarding 1L-CPC, the gradient was interrupted after 140 minutes, since the separation of the two first classes occurred in the first part, and the remaining compounds were collected by stationary phase extrusion.

Barbera and Nebbiolo TAEs were injected separately since their anthocyanin profiles is different. Therefore, differences in fractions collection were applied and a total of 8 and 7 fractions were collected for Barbera and Nebbiolo, respectively.

To fractionate acetylated and coumaroylated anthocyanins, a further purification was needed to achieve a satisfactory level of purity and preparative HPLC was carried out. Chromatographic peaks were collected manually, and the collected fractions were evaporated and freeze-dried twice to avoid the presence of solvents, and stored at −20 °C Purity and composition of fractions were checked with the HPLC-DAD system.

### Anthocyanins-Protein binding test

#### BSA test

The bovine serum slbumin (BSA) method for predicting astringency of tannins was modified for the analysis of anthocyanins. The method was described by Boulet *et al*.^53^ for wine and was modify in order to achieve repeatability of results, testing different amount of Bovine Serum Albumin Fraction V (Sigma–Aldrich, Saint Quentin Fallavier, France, 2 and 4 mg/mL), and of anthocyanins extract (0.5, 1 and 2 mg/L) and reaction time (15, 30, and 40 minutes) and waiting time after centrifugation (0, 15, 30 minutes). Variation between treated and untreated samples were checked by spectrophotometric lecture at λ = 520 nm after 10 dilutions with 2% HCl solution (V-630 UV–vis spectrophotometer, JASCO, Japan) and direct HPLC-DAD injection. Finally, good coefficient of variation (<5%) was achieved using the following protocol. Barbera TAE and CPC fractions GF, AF, and CF were dissolved in wine-like solution (12% ethanol, 4 g/L tartaric acid, 3.5 pH) at a concentration of 1 g/L and centrifuged at 13500 *g* for 10 minutes to eliminate all the insoluble material. BSA was dissolved at concentration of 4 mg/mL in pH 4.9 buffer solution and 0.5 ml were added to 2 ml anthocyanin solution samples (BSA) and buffer solution without BSA were added to 2 ml anthocyanin solution samples (control). Samples were left under slight agitation for 30 minutes before being centrifuged 13500* g* for 5 minutes. The supernatant was filtered through a 0.45 µm filter and inject in HPLC-DAD system as described before for quantitative analysis. Each analysis was performed in triplicate. Reactions with BSA were then measured as the difference (delta) between the sample without BSA (control) and sample with BSA (BSA).

#### Saliva test

Saliva collection was performed from 18 volunteers (6 males and 12 females aged 20 to 35 years old) from 10 to 12 a.m. to follow circadian rhythm^54^. Volunteers were asked to avoid eating and drinking beverages for at least one hour before sampling. Saliva was collected in 5 ml Eppendorf tubes, pooled together and immediately stored at −20 °C before freeze-drying. Lyophilized saliva was dissolved at 10 mg/L -corresponded to one/third concentration as reported by Ma *et al*.^55^ in phospate buffer at pH 6.8 and centrifugated 8000 *g* for 5 min at 4 °C by a Jouan MR22 refrigerated centrifuge and the supernatants used as salivary protein sample. The method was that of Schwarz and Hoffman, 2008^9^ with some modifications. Barbera TAE, and CPC fractions GF, AF, and CF were dissolved 1 mg/ml in wine-like solution (12% ethanol, 4 g/L tartaric acid, pH 3.5). A target compounds solution (300 μL) was mixed with 700 μL of prepared saliva sample or phosphate buffer as control and incubated at 37 °C for 5 min. After incubation, an aliquot (400 μL) of the mixture was moved to a 3k Da centrifugal filter (Amicon Ultra-0.5 Centrifugal Filter 3k Devices, Merck Millipore) and centrifuged at 18,000 rpm for 5 min at 37 °C. The filtrate in the bottom was injected into the HPLC-DAD system for quantitative analysis. Each analysis was performed in triplicate. Reactions with saliva were then measured as the difference between the sample without salivary protein (control) and sample with salivary protein (saliva).

#### Statistical analysis

Statistical analyses were carried out using R Statistics software version 3.4.0 (R Core Team, 2017) for one-way analysis of variance (ANOVA) and correlation. Levene’s and Shapiro-Wilk’s tests were used for assessing the homogeneity of variance and normality of ANOVA residuals, respectively. Correlation between anthocyanins decrease (treated-untreated samples as delta) and anthocyanins concentration was carried out depending on anthocyanidins substitution and anthocyanins esterification. Shapiro-Wilk’s test for normality of distribution was carried out and correlation was calculated by Pearson or Spearman correlation formula if normally or not normally distributed, respectively.

### Sensory Analysis

Sensory analyses were conducted in a tasting room at our oenology research unit (ISVV, France) corresponding to the ISO 8589:2007 standards for this type of equipment (sound insulation, constantly regulated temperature).

#### Panel selection

All of the judges came from ISVV and are experienced with wine tasting. Judges were tested for determine if they can determine the interested sensory properties, i.e. basic taste found in wine, and astringency by tasting standard solutions: aluminium sulphate 2 g/L for astringency, quinine sulphate 15 mg/L for bitterness, tartaric acid 5 g/L for acidity, catechin 1 g/L for astringency and bitterness together. In order, two test were carried out: triangular test and identification of the the descriptors. In triangular test, equal number of the six possible combinations (ABB, BAA, AAB, BBA, ABA, and BAB, where A is the wine-like solution and B is the wine-like spiked with the molecule of interest) were proposed and judges were asked to recognize the different sample in the series. For identification test, the four spiked wine-like solutions were proposed and was asked to identify and describe the in-mouth sensation perceived. Judges who could not recognize the descriptors were not include in the panel. The final panel consisted of 18 judges, 12 females and 6 males aged 20-45.

#### In-mouth detection thresholds

In all experiments, black glasses filled with 8 mL of solution were labelled with three-digit random codes and presented to the panellists in random order for each presentation (following the scheme AAB, ABA, BAA where A is the wine-like solution and B is the wine-like spiked with the extract/fraction of interest), and presentation were randomized as well so to have an equal number of the possible combinations. Solutions, at room temperature, were presented in black glass in order to avoid colour influence, and judges were also instructed to spit in a black glass to avoid seeing the difference meanwhile expectoration. Each judge was asked to sip the total glass volume, for avoiding differences given by the quantity tasted. Between each sample, judges were asked to take a 30 seconds rest, and water and cracker were provided for each presentation. In-mouth detection thresholds of the Barbera total anthocyanins extract, and CPC fractions GF, AF, and CF in wine-like solution (12% ethanol, 4 g/L tartaric acid, pH 3.5) were estabilished. The detection threshold was determined using the three alternative forced-choice presentation method 3-AFC (ISO 13301:2002) at concentration representative of the real wine concentration, i.e for total anthocyanins from 62.5 to 2000 mg/L, glucoside fraction from 31.25 to 1000 mg/L, for both acetylated and cinnamoylated fractions from 3.125 to 100 mg/L. A dilution factor of 2 for 6 total presentations was applied. The concentration were choosen because of the content of anthocyanins in wine and after a preliminary essay (triangular test, n = 7) as suggested by Meilgaard *et al*.^56^. Four tasting sessions were performed for total anthocyanins extract, glucoside, acetylated and cinnamoylated fractions, respectively. In each session, samples were presented following increasing concentration for each presentation as reported above. Judges were asked to specify one or more descriptors belonging to in-mouth properties that allowed the sample to be discriminated. The corresponding detection threshold was calculated as best estimated threshold (BET)^56^. The individual BET was determined as the geometric mean of the highest concentration missed and the next higher concentration. For judges who were correct at the lowest concentration, their individual BET was estimated as the geometric mean of the lowest concentration and the hypothetical next lower concentration that would have been given. For judges who failed to correctly identify the highest concentration, their individual BET was estimated as the geometric mean of the highest concentration tested and the next higher concentration that would have been given had the series been extended. The group BET was calculated as the geometric mean of the individual BET. Standard deviation log_10_ provided a measure of the group’s variation.

## Electronic supplementary material

Supplementary information
