# Supplementary material for: Chemical and sensorial investigation of in-mouth sensory properties of grape anthocyanins
Source: Sci Rep. 2018 Nov 20;8:17098. doi: 10.1038/s41598-018-35355-x (PMC6243997; doi:10.1038/s41598-018-35355-x)
Supplement: Supplementary file 1 — Supplementary information [file 41598_2018_35355_MOESM1_ESM.pdf]

Supplementary Information

Chemical and sensorial investigation of in-mouth sensory properties of grape anthocyanins

M.A. Paissoni, P. Waffo-Teguo, W. Ma, M. Jourdes, L. Rolle, P-L. Teissedre

Reference	*Variety*	*Area*	*Vintage*	*Total Anthocyanins Content*				*Method*	*Glucoside*	*Acetylated*	*Cinnamoylated*
				**Grape**	***Wine***				**mg/L Month (n)**	**mg/L Month (n)**	**mg/L Month (n)**
				**mg/Kg**	**mg/L Month (n)**	**mg/L Month (n)**	**mg/L Month (n)**				
Mazza et al., 1999 (18)	Cabernet Franc	Okanagan Valley (United States)	1996	826	420(1)	316 (8)	232 (14)	Spect*	-	-	-
			1997	1048	469(1)	337(8)					
	Merlot		1996	981	412(1)	371 (8)	279 (14)				
			1997	1047	455(1)	338(8)					
	Pinot Noir		1996		340(1)	280 (8)	223 (14)				
			1997		219(1)	171(8)					
Gonzales-Neves et al., 2004 (19)	Tannat	Uruguay	2001	1459	551(2)	-	-	Spect*	-	-	-
			2002	3632	952(2)						
	Cabernet Sauvignon		2001	1079	349(2)						
			2002	1939	563(2)						
	Merlot		2001	708	226(2)						
			2002	1516	402(2)						
García-Falcón et al., 2007 (20)	Mencia	Ribeiro (Spain)	-	-	205 (End MLF)	129 (3)	57 (12)	HPLC-DAD	156 (end MLF); 98 (3); 40(12)	25 (end FML); 17 (3); 8 (12)	24(end MLF); 14(3); 3(12)
	Brancellao				66 (End MLF)	53 (3)	10 (12)		60 (end MLF); 48 (3); 5(12)	6 (end FML); 5 (3); 1 (12)	1(end MLF); 0.8(3); n.q.(12)
Perez-Magariňo et al., 2004 (21)	Tinto Fino	Ribeira del Duero (Spain)	-	Different Ripeness	343 (End AF)	-	-	HPLC-DAD	304	16	23
					401 (End AF)				358	23	20
					367 (End AF)				322	20	25
	Cabernet Sauvignon			Different Ripeness	561 (End AF)				375	153	33
					576 (End AF)				401	151	24
					593 (End AF)				397	159	37
Ferrandino et al., 2012 (22)	Barbera	Piedmont (Italy)	2006	1220	-	-	-	HPLC-DAD	*79.2%*	*10.1%*	*10.7%*
			2007	1264					*78.9%*	*11.3%*	*9.8%*
	Nebbiolo		2006	668					*92.2%*	*2.5%*	*5.3%*
			2007	828					*90.9%*	*4.5%*	*4.6%*
	Cabernet Sauvignon		2006	1474					*67.7%*	*25.4%*	*6.9%*
			2007	1380					*67.3%*	*24.7%*	*8.0%*
Cagnasso et al., 2008 (23)	Nebbiolo	Piedmont (Italy)	2000	494	184(0.5)	-	-	Spect* (Total Anthocyanins)	*90.7%*	*8.7%*	*0.6%*
			2001	428	195(0.5)				*93.6%*	*5.8%*	*0.6%*
			2000		200(0.5)				*93.2%*	*6.7%*	*0.1%*

			2001		231(0.5)			HPLC-UV (percentages)	*92.0%*	*6.9%*	*1.1%*
			2000		149(0.5)				*93.0%*	*5.7%*	*1.3%*
			2001		168(0.5)				*72.9%*	*24.9%*	*2.2%*
	Barbera		2000	981	454(0.5)				*78.8%*	*21.0%*	*0.2%*
			2001	1131	707(0.5)				*81.9%*	*12.6%*	*5.5%*
			2000		519(0.5)				*75.0%*	*24.7%*	*0.3%*
			2001		547(0.5)				*76.7%*	*19.0%*	*4.3%*
Lingua et al., 2016 (24)	Syrah	San Juan (Argentina)		1709.21	334.65 (End AF)	154.85 (FW)			456.97 (Grapes); 185.48 (End AF); 92.15 (FW)	909.03 (Grapes); 107.42 (End AF); 52.59 (FW)	340.76 (Grapes); 41.75(End AF); 10.05 (FW)
	Merlot		-	757.81	271.68 (End AF)	72.76 (FW)	-	HPLC-DAD-MS	344.17 (Grapes); 171.56 (End AF); 50.87 (FW)	329.95 (Grapes); 76.03 (End AF); 16.49 (FW)	83.22 (Grapes); 24.09 (End AF); 5.39 (FW)
	Cabernet Sauvignon			1025.7	119.79 (End AF)	96.52 (FW)			355.33 (Grapes); 85.06 (End AF); 70.19 (FW)	584.16(Grapes); 25.19 (End AF); 22.36 (FW)	86.05 (Grapes); 9.50 (End AF); 3.97 (FW)
Alcalde Eon et al., 2006 (25)	Tannat	Cerro Chapeu (Uruguay)	2003	nd	762.4 (3)[†]				*45.20%*	*27%*	*14.10%*
	Caladoc		2003	nd	469.1 (3)[†]				*42.60%*	*28.70%*	*16.40%*
	Marselan		2003	nd	445.4 (3)[†]	-	-	HPLC-DAD-MS	*40.60%*	*30.90%*	*12.30%*
	Marzemino		2003	nd	497.1 (3)[†]				*33.70%*	*33.60%*	*9%*
	Chevenasco		2003	nd	214.5 (3)[†]				*46.90%*	*10.30%*	*8%*
García-Marino et al., 2010 (26)	Tempranillo	La Rioja (Spain)	nd	nd	993.63 (1)	608.95(13)	310.61 (27)	HPLC-DAD	794.54(1); 452.27(13); 171.50(27)	163.23(1); 121.04(13); 111.29(27) [‡]	
	Graciano		nd	nd	1217.83 (1)	668.78(13)	380.77 (27)		942.01(1); 485.76(13); 230.37 (27)	239.64(1); 147.39(13); 120.64(27) [‡]	
Fanzone et al., 2012 (27)	Malbec	Mendoza (Argentina)	2010	nd	1044.5[†]; 551.2[§] (End MLF)				405.8 (*69.1%*)	97.8 (*16.7%*)	47.6 (*8.1%*)
	Bonarda		2010	nd	739.8[†]; 285.5[§] (End MLF)			Spect[*] (Total Pigments)	212.8 (*69.2%*)	43.9 (*14.3%*)	28.8 (*9.4%*)
	Cabernet Sauvignon		2010	nd	681.8[†]; 269.6[§] (End MLF)	-	-	HPLC-DAD-MS (Monomeric Anthocyanins)	182.8 (*63.2%*)	72.1 (*25%*)	14.7 (*5%*)
	Merlot		2010	nd	644.1[†]; 273.7[§] (End MLF)				183.8 (*62.5%*)	66.3 (*22.5%*)	23.6 (*13.5%*)
	Shiraz		2010	nd	301.4[†]; 168.3[§] (End MLF)				97.4 (*54.8%*)	47.1 (*26.5%*)	23.8 (*13.5%*)

	Tempranillo		2010	nd	717.6†; 306.5§ (End MLF)				242.9 (*75.8%*)	27.7 (*8.7%*)	35.9 (*11.2%*)
Ginjom et al., 2010 (28)	Shiraz	New South Wales (Australia)	2003	-	86(36)	-	-	Spect*	-	-	-
			2004		289(24)						
			2005		299(12)						
	Cabernet Sauvignon	New South Wales (Australia)	2003		160(36)						
			2004		267(24)						
		Queensland (Australia)	2005		189(12)						
	Merlot	New South Wales (Australia)	2003		137(36)						
			2004		122(24)						
		South Australia (Australia)	2005		211(12)						
Romero-Cascales et al., 2005 (29)	Monastrell	Jumilla (Spain)	2003	1110	361(0.5)	-	-	HPLC-DAD-MS	301.2 (*83%*)	59.8 (*17%*)	
	Cabernet Sauvignon			751.9	354.2(0.5)				211.84 (*58.6%*)	142.36 (*41.4%*)	
	Syrah			914.9	350.8(0.5)				216.75 (*61.4%*)	134.05 (*38.6%*)	
	Merlot			582.1	225.5(0.5)				146.8 (*64%*)	78.6 (*36%*)	
Chira et al., 2011 (30)	Cabernet Sauvignon	Bordeaux (France)	1978	-	15.31	-	-	Spect*	-	-	-
			1988		46.38						
			2000		64.31						
			2005		123.38						
	Merlot	Bordeaux (France)	1979		9.63						
			1988		21.88						
			1998		17.94						
			2003		32.38						

Table S1 Overview of anthocyanin content in winegrapes and monovarietal wines from different areas, vintages, and aging times. Footnotes and abbreviations legends: * Indicates spectrophotometric measure, as total anthocyanins; *Italic* indicates % of glucoside, acetylated and cinnamoylated on total anthocyanins; † indicates total pigment content; ‡ indicates acylated as sums of acetylated, *p*-coumaroylated, and caffeoylated derivatives; § monomeric anthocyanins analysed through HPLC-DAD. MLF = malolactic fermentation; AF= alcoholic fermentation.
